# Nano-drops of biomimetic macromolecular drugs based on macrophage membranes for targeted treatment of fundus diseases

**DOI:** 10.1016/j.mtbio.2026.103577

**Published:** 2026-08-19

**Authors:** Yongguo Xiang, Jiaojiao Kou, Huijie Cao, Yuhui Zhang, Hong Cheng, Tu Wang, Jialing Jiang, Shuren Pan, Juan Kang, Yuanfu Ding, Wenjuan Wan, Shijie Zheng, Man Li, Xiaobei Huang, Ke Hu

**Affiliations:** a Department of Ophthalmology, The First Affiliated Hospital of Chongqing Medical University, Chongqing Key Laboratory for the Prevention and Treatment of Major Blinding Eye Diseases, Chongqing Eye Institute, Chongqing, 400010, China; b Department of Ophthalmology, 363 Hospital, Chengdu, 610041, China; c Chongqing Institute of Green and Intelligent Technology, Chinese Academy of Sciences, Chongqing, 400714, China; d CQMU - University of Leicester Joint Institute, Chongqing Medical University, Chongqing, 401331, China; e Changdu People's Hospital of Xizang, Changdu, 854085, China; f School of Medical and Information Engineering, Scientific Research Center, Gannan Medical University, Ganzhou, 341000, China

**Keywords:** Eyedrops, Non-invasive, Ocular barriers, Ranibizumab, Choroidal neovascularization

## Abstract

Ocular fundus diseases—including age-related macular degeneration and diabetic retinopathy—are leading causes of irreversible vision loss worldwide. Current macromolecular therapies require frequent intravitreal injections, which increase patient burden and complication risks. To address these limitations, we engineered a macrophage membrane-biomimetic nanovesicle encapsulating ranibizumab (designated M@R) for noninvasive ocular delivery and selective targeting of inflammatory lesions in the posterior segment. *In vitro* studies demonstrated that M@R retained potent cytokine-scavenging activity against key pro-inflammatory mediators, and the macrophage membrane coating significantly enhanced transcytosis-mediated transport of ranibizumab across ocular epithelial barriers. *Ex vivo* and *in vivo* studies confirmed that M@R is capable of noninvasive traversal of ocular barriers, achieving targeted delivery to inflammatory lesions within the fundus. In a laser-induced choroidal neovascularization (CNV) mouse model, topical ocular administration of M@R significantly reduced both the area and thickness of CNV, attributable to its dual anti-inflammatory and anti-VEGF activities. Although multiple dosing was required to achieve therapeutic efficacy comparable to that of a single intravitreal ranibizumab injection, M@R exhibited markedly improved safety profiles, with no observable local ocular toxicity or systemic adverse effects following repeated administration. Collectively, these findings establish macrophage membrane-coated nanovesicles as a promising platform for barrier-penetrating, inflammation-targeted delivery of biologics in the treatment of fundus diseases.

## Graphical abstract

**Figure:**
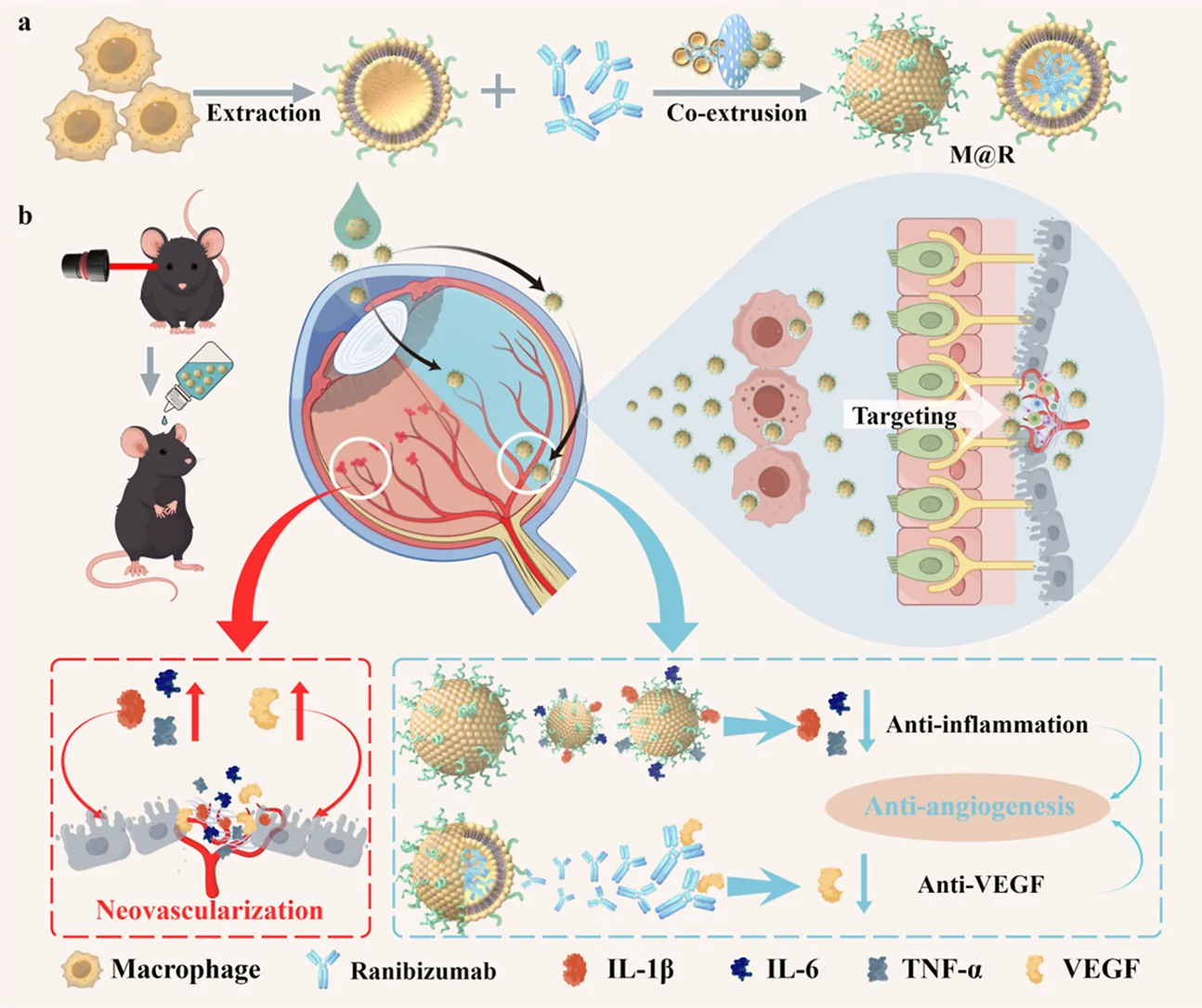

## 1 Introduction

Ocular fundus diseases, including age-related macular degeneration (AMD), diabetic retinopathy (DR), and retinal vein occlusion (RVO), are leading causes of irreversible vision loss worldwide [[1], [2], [3], [4]]. As the global population continues to age, the prevalence of these conditions is expected to rise, imposing substantial societal and healthcare burdens [1]. Macromolecular biologic agents have become pivotal in treating such diseases, with approved therapeutics—including ranibizumab, conbercept, and aflibercept—widely used for neovascular AMD (nAMD), diabetic macular edema (DME), DR, and RVO [[5], [6], [7]]. However, due to their high hydrophilicity and large molecular size, these macromolecules cannot effectively penetrate ocular barriers via conventional topical administration [[8], [9], [10], [11], [12], [13]]. Consequently, intravitreal injection remains the primary route for delivering macromolecular therapeutics to the posterior segment of the eye. Moreover, the limited stability and short intraocular half-life of many biologic agents often necessitate frequent injections [14], which increases the burden on patients and healthcare systems while elevating the risk of injection-related complications such as cataract, endophthalmitis, hemorrhage, and retinal detachment [15,16]. Therefore, the development of a drug delivery system capable of non-invasive, safe, and efficient transport of macromolecular drugs to the posterior eye segment holds significant clinical relevance.

Cell membrane-biomimetic nanotechnology integrates the intrinsic biological properties of natural cell membranes with the tunable characteristics of synthetic nanomaterials. This strategy enhances biocompatibility, prolongs systemic circulation, and enables targeted drug delivery [[17], [18], [19]]. Macrophage membrane-coated nanoplatforms inherit the inflammation targeting behavior of macrophages and have been shown to efficiently cross biological barriers, including the blood-brain barrier (BBB) and the blood-retinal barrier [[20], [21], [22], [23]]. Evidence suggests that this barrier penetration occurs primarily through receptor-mediated transcytosis [24,25]. Notably, the corneal and conjunctival epithelia, along with their tight-junction complexes, constitute the principal ocular surface barrier and exhibit considerable structural similarity to the BBB [8,26]. Based on this resemblance, we hypothesized that macrophage membrane-biomimetic carriers could similarly facilitate traversal of ocular surface barriers.

Chronic inflammation drives the pathogenesis and progression of many fundus diseases. In conditions like nAMD and DR, lesion sites show elevated levels of inflammatory cytokines and chemokines. These molecules recruit and activate macrophages within retinal and choroidal tissues. Once activated, macrophages secrete abundant inflammatory mediators, which amplify local inflammation and create a self-perpetuating feed-forward loop [[27], [28], [29], [30]].

Prior studies indicate that macrophage membrane-coated nanoparticles preserve the inflammation-homing ability of their parent cells. After systemic or intravitreal administration, these particles can cross biological barriers and accumulate at inflammatory fundus lesions [23,31]. Their membrane surface retains receptors such as TNF-α-R, IL-6-R, and IL-1β-R. These receptors can bind and sequester inflammatory cytokines in the pathological microenvironment [20,32,33]. This process may mitigate local inflammation and disrupt the inflammatory amplification cycle, thereby potentially enhancing therapeutic outcomes. Accordingly, we postulated that a macrophage membrane-based biomimetic carrier could selectively target ocular inflammatory sites and neutralize key inflammatory mediators.

**Scheme 1:**
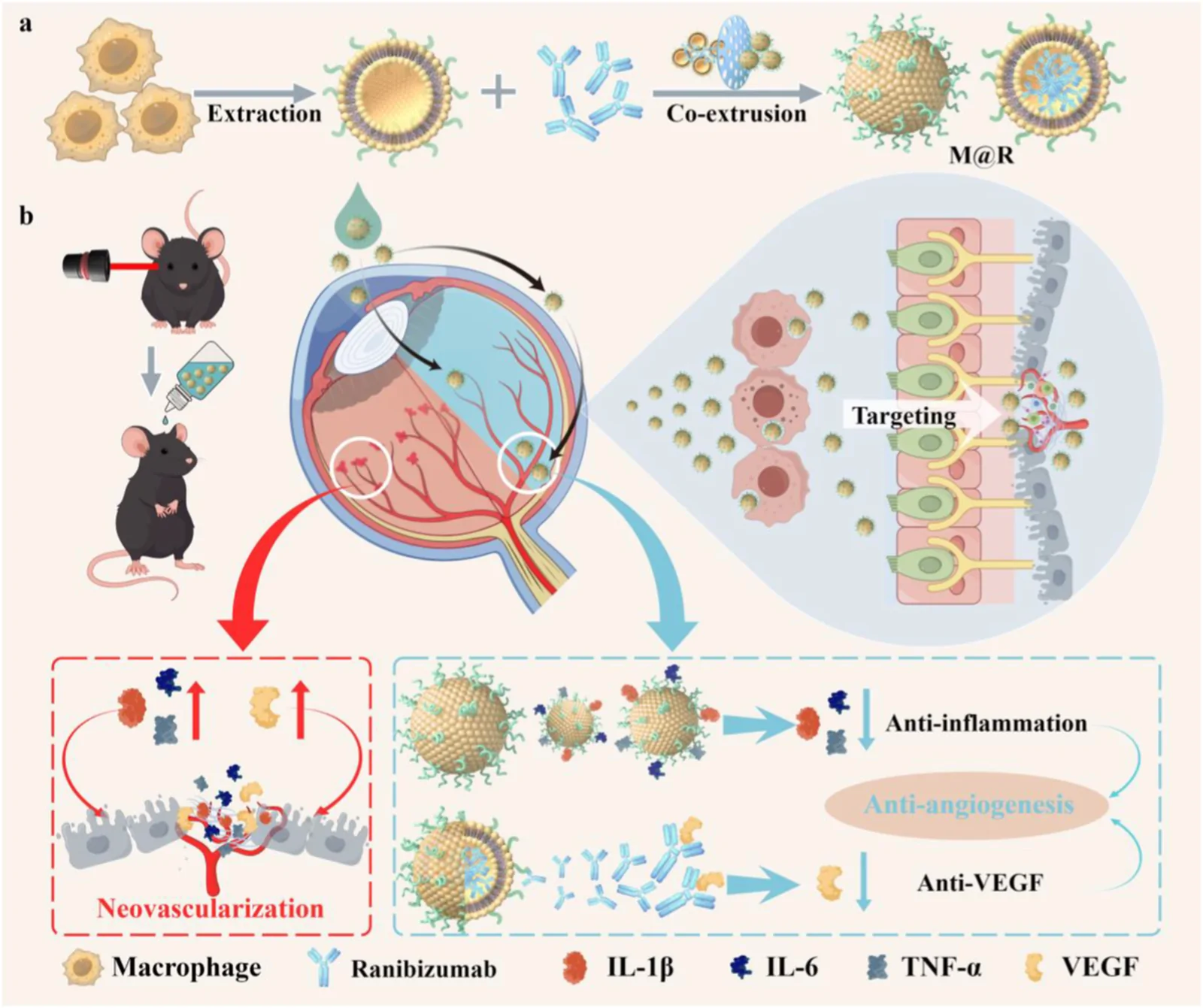
Schematic illustration of macrophage membrane-coated ranibizumab (M@R) for noninvasive targeted therapy of neovascular age-related macular degeneration (nAMD). **(a)** Preparation of M@R. **(b)** Noninvasive targeting and inhibition of neovascularization by M@R eye drops via cytokine and VEGF neutralization.

To achieve noninvasive, targeted delivery of macromolecular therapeutics to fundus inflammatory lesions, this study developed a macrophage membrane-biomimetic nanodrop encapsulating ranibizumab (M@R; Scheme 1). The formulation was prepared by co-extrusion of macrophage membranes with ranibizumab. We demonstrated that the macrophage membrane coating enhanced transcellular transport of ranibizumab, promoting its penetration across the cornea and conjunctiva-sclera-choroid pathway, ultimately achieving targeted delivery to inflammatory sites in the fundus. Concurrently, inflammatory cytokine receptors retained on the membrane conferred cytokine adsorption capacity. In a laser-induced choroidal neovascularization (CNV) mouse model, topical administration of M@R achieved selective accumulation at CNV lesions. Through combined cytokine adsorption and anti-VEGF activity, M@R significantly reduced both CNV area and thickness. Although repeated instillation was required, its therapeutic efficacy was comparable to that of intravitreal ranibizumab injection. Furthermore, prolonged topical application of M@R produced no apparent adverse effects in the cornea, retina, or major systemic organs, indicating favorable biocompatibility. Collectively, this macrophage membrane biomimetic strategy provides a promising platform for noninvasive, targeted topical delivery of ocular macromolecular therapeutics.

## 2 Results and discussion

### 2.1 Synthesis and characterization of M@R

**Fig. 1:**
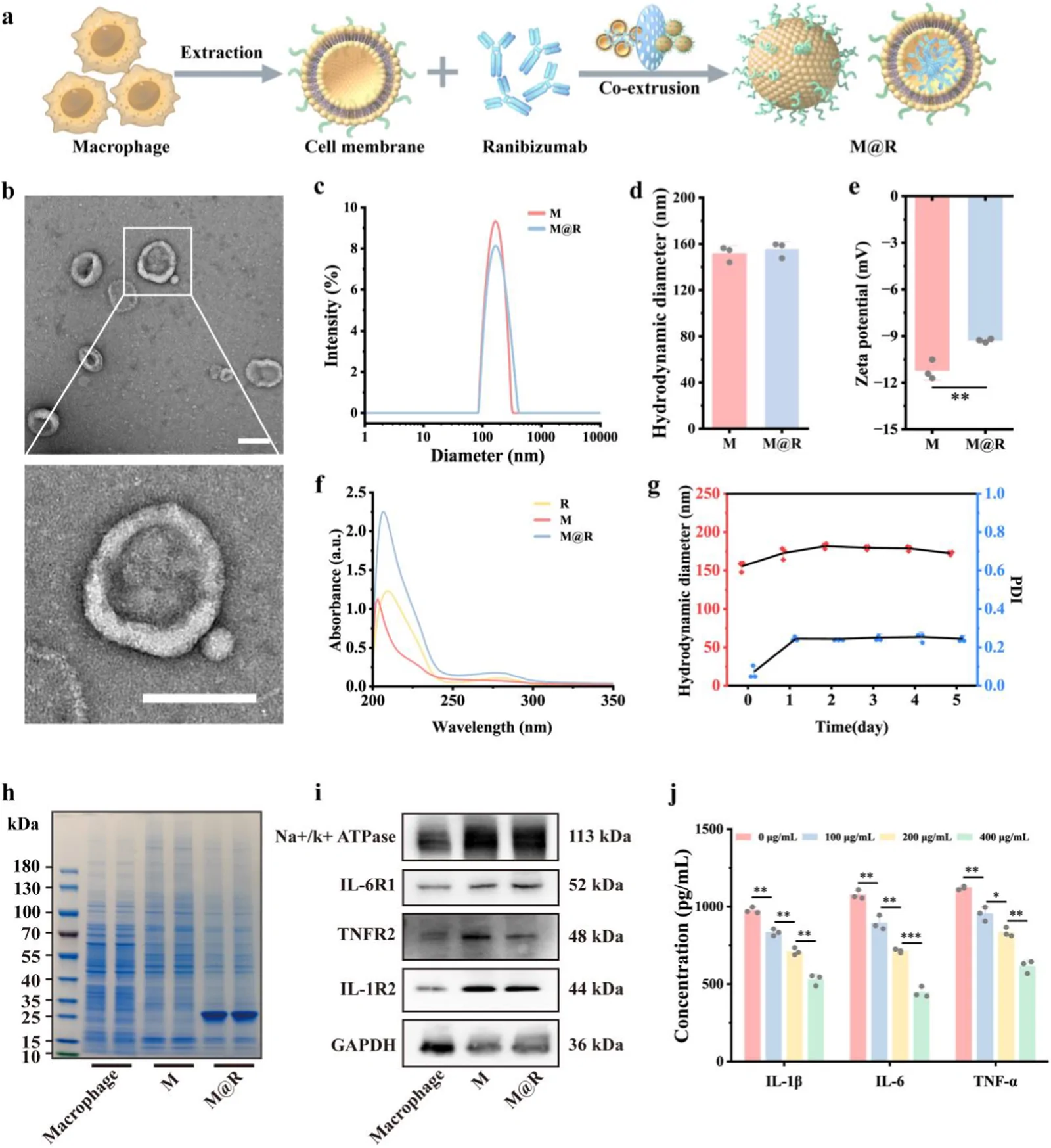
**Synthesis and characterization of M@R. (a)** Schematic of M@R preparation. **(b)** TEM images of M@R. Scale bar, 100 nm. **(c)** Particle size distributions of M and M@R. **(d)** Hydrodynamic diameters of M and M@R (n = 3). **(e)** Zeta potentials of M and M@R (n = 3). **(f)** UV–Vis absorbance spectra of R, M, and M@R. **(g)** Stability of M@R in PBS assessed by monitoring particle size and PDI over time (n = 3). **(h)** Protein profiles of macrophages, M, and M@R analyzed by SDS-PAGE. **(i)** Western blot analysis of TNFR2, IL-1R2, IL-6R, GAPDH, and Na^+^/K^+^ ATPase in macrophages, M, and M@R. **(j)** ELISA quantification of IL-1β, IL-6, and TNF-α following the indicated treatments (n = 3). Data are presented as mean ± SD. Statistical analysis was performed using independent-samples T test. *p < 0.05; **p < 0.01; ***p < 0.001.

The extracted macrophage membranes were processed through a liposome extruder to prepare macrophage membrane vesicles (M). Macrophage membrane-coated ranibizumab (M@R) was fabricated by co-extruding macrophage membranes with ranibizumab at a mass ratio of 1:1 using the same extrusion system (Fig. 1a). The encapsulation efficiency and drug loading capacity of ranibizumab in M@R were evaluated with a BCA protein assay kit, yielding values of 31.8% and 15.9%, respectively. Transmission electron microscopy images revealed a core-shell structure for M@R, with a single-layer cell membrane coating its surface (Fig. 1b). Dynamic light scattering analysis indicated that M@R (diameter: 160 nm; PDI: 0.05) exhibited a narrow size distribution. Although its diameter was slightly larger than that of M (156 nm; PDI: 0.14) (Fig. S1), the difference was not statistically significant (151.80 ± 6.63 nm *vs.* 155.43 ± 6.67 nm, *p* = 0.540; Fig. 1c and d). In contrast, the zeta potential of M@R was significantly less negative than that of M (−9.27 ± 0.12 mV *vs.* −11.2 ± 0.62 mV, *p* = 0.006; Fig. 1e). UV-Vis spectroscopy was used to examine the absorption profiles of R, M, and M@R in the 200–350 nm range. Characteristic peaks were observed for both R and M@R (Fig. 1f), confirming successful encapsulation of R within the membrane vesicles. Moreover, M@R maintained a consistent particle size and polydispersity index for at least 5 days when stored in PBS at 4 °C (Fig. 1g, Fig. S2).

Following successful preparation of M@R, the protein profiles of macrophages, M, and M@R were analyzed by SDS-PAGE. As shown in Fig. 1h–M and M@R shared similar protein patterns that were distinct from whole macrophage lysates, consistent with removal of intracellular proteins during membrane isolation. Compared with M, M@R displayed characteristic bands corresponding to R, supporting successful loading. Macrophage membrane-biomimetic nanocarriers have been reported to exhibit inflammation targeting and cytokine adsorption, which depends on preservation of functional membrane proteins on the macrophage membrane surface [31,32]. As shown in Fig. 1i, western blot analysis revealed that the membrane marker Na^+^/K^+^ ATPase was highly expressed in both the M and M@R groups, confirming successful extraction of macrophage membrane fractions. Concurrently, robust bands corresponding to cytokine receptors, including TNF-αR2, IL-1βR, and IL-6R, were observed in both groups, indicating that functional membrane proteins were largely preserved during the isolation process. However, faint signals of the cytosolic marker GAPDH were also detected in both preparations, suggesting that while the majority of cytoplasmic proteins had been removed, trace contamination remained within the membrane pellets. The strong enrichment of Na^+^/K^+^ ATPase and the retention of multiple cytokine receptors demonstrate the feasibility of our membrane extraction protocol and confirm that the biomimetic carrier retains key surface proteins essential for inflammatory homing and cytokine neutralization. The presence of these receptors aligns with the proposed strategy of using macrophage-derived membranes to intercept pro-inflammatory mediators in the lesion microenvironment. Although the weak GAPDH bands indicate only minimal cytoplasmic contamination; such residual impurities might interfere with downstream quantitative assays or *in vivo* specificity, particularly when precise receptor density or protein quantification is required. The likely cause of this minor contamination is the absence of additional purification steps, such as ultracentrifugation and repeated washing cycles, in the current protocol. Therefore, future optimization should incorporate these measures to further enhance membrane purity and reduce non-specific backgrounds, thereby improving the reliability of subsequent functional evaluations.

The cytokine-adsorbing capacity of M@R was further evaluated *in vitro*. Incubation of M@R with TNF-α, IL-1β, and IL-6 led to a dose-dependent decrease in these cytokines (Fig. 1j). Similarly, coculture with M@R significantly reduced TNF-α, IL-1β, and IL-6 levels in supernatants of M1-polarized macrophages (Fig. S3). Collectively, these results indicate that macrophage membrane vesicles successfully encapsulated ranibizumab while preserving key membrane proteins responsible for cytokine adsorption.

### 2.2 M@R promotes transcellular delivery of ranibizumab *in vitro* and analysis of its mechanism

The ocular biocompatibility of M@R was first evaluated by assessing its cytotoxicity toward HCE-T and ARPE-19 cells using the CCK-8 assay and live/dead staining. As shown in Fig. S4, incubation with M or M@R at concentrations up to 40 μg mL^−1^ for 24 h did not significantly affect cell viability. Since high concentrations of membrane material interfered with absorbance readings in the CCK-8 assay, live/dead staining was further performed at 1 mg mL^−1^. After 24 h of treatment, no notable increase in PI-positive cells was observed in the M or M@R groups compared with the untreated control (Fig. S4), supporting favorable biocompatibility of M@R.

**Fig. 2:**
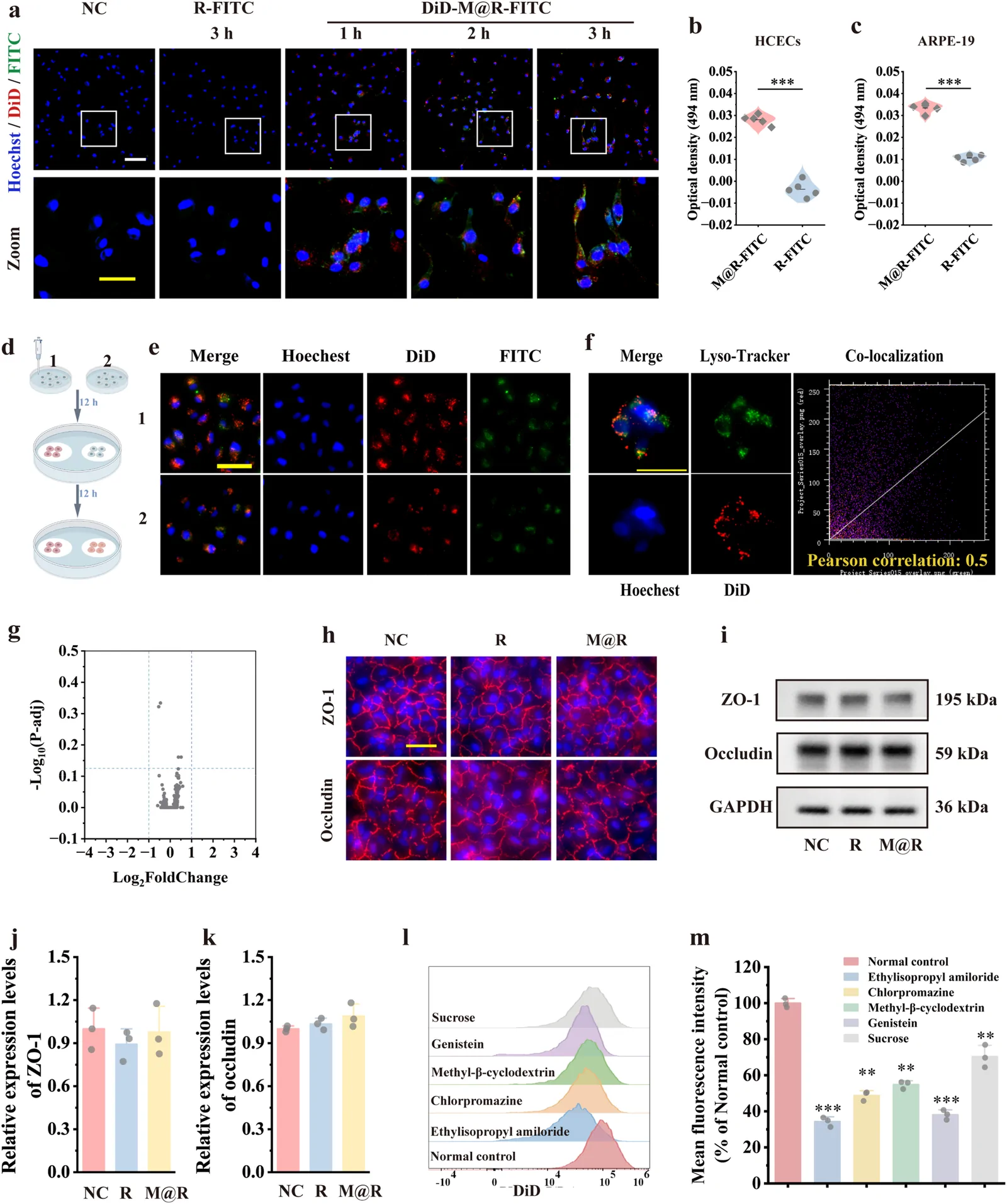
**M@R promotes the intracellular internalization of ranibizumab *in vitro*. (a)** Representative fluorescence images of R-FITC and DiD-M@R-FITC uptake by HCECs at various time points. The Transwell chamber was used to construct the *in vitro* HCEC cell barrier model **(b)** and the RPE cell barrier model **(c)**, and the average absorbance of R-FITC and M@R-FITC in the transwell inferior chamber at a wavelength of 494 nm (n = 5). **(d)** Scheme illustrating the intercellular transport of M@R between HCECs. The cells (1) were cultured with DiD-M@R-FITC for 12 h, washed with PBS, the cells (1 and 2) were cultured together for 12 h, and followed by imaging. **(e)** Fluorescent images of HCECs were acquired to assess the intracellular transport of DiD-M@R-FITC. **(f)** Co-localization images of lysosomes (Green) and M@R (Red) in HCECs, along with Pearson coefficient analysis. **(g)** Volcano plots showing differentially expressed genes (DEGs) from HCECs to PBS or M@R treatment. **(h)** Immunofluorescence images of ZO-1 and occludin indicating the distribution of tight junctions in HCECs after treatment with PBS, R, or M@R. **(i)** Western blot analysis indicated the expression levels of tight junction-associated proteins ZO-1 and occludin in HCECs after treatment with PBS, R, or M@R. Relative expression levels of ZO-1 **(j)** and occludin **(k)** protein in HCECs treated with PBS, R, or M@R (n = 3). **(l)** Evaluation of the effect of different endocytosis inhibitors on M@R entry into HCECs using flow cytometry. **(m)** Inhibition of endocytosis effects by sucrose, chlorpromazine, mβ-cyclodextrin, genistein, and amiloride on M@R uptake in HCEC (n = 3). Data are presented as mean ± SD. Statistical analysis was performed using independent-samples T test or one-way ANOVA with multiple comparisons corrected by the Bonferroni test. **p < 0.01; ***p < 0.001. Scale bars: 100 μm (white), 50 μm (yellow).

Next, we investigated how M@R promotes the ocular uptake and transcellular delivery of R. FITC and DiD were used to label R and the macrophage membrane (M), respectively. While negligible green fluorescence was detected in HCE-T after 3 h of incubation with R-FITC, strong red (DiD) and green (FITC) signals were observed in cells treated with DiD-M@R-FITC, and the fluorescence intensity increased over time (Fig. 2a, Fig. S5). A similar enhancement was observed in ARPE-19 and HUVECs (Figs. S6 and S7), indicating that macrophage membrane coating markedly improves the cellular uptake of R by ocular cells.

To evaluate the ability of M@R to cross cellular barriers, HCE-T or ARPE-19 cells were cultured on Transwell inserts until a confluent monolayer was formed. After adding M@R-FITC or R-FITC to the upper chamber, absorbance in the lower chamber was measured after 12 h. The M@R-FITC group showed significantly higher absorbance than the R-FITC group for both cell types (Fig. 2b and c), demonstrating that macrophage membrane coating markedly enhances trans-barrier transport of R.

We further examined whether M@R undergoes transcytosis according to a previously reported protocol [34]. Donor HCE-T cells were incubated with DiD-M@R-FITC for 12 h, washed, and then cocultured with untreated recipient HCE-T cells in fresh medium for another 12 h (Fig. 2d). Fluorescence microscopy revealed both red and green signals in recipient cells (Fig. 2e), suggesting that M@R can be transported via transcytosis. To evaluate vesicle integrity, we analyzed the colocalization of DiD (membrane) and FITC (ranibizumab) during uptake and transcellular transport. A high Pearson's correlation coefficient (>0.7, Figs. S8 and S9) was maintained, indicating that the vesicles remained largely intact throughout the process. Moreover, lysosomal colocalization analysis showed limited overlap between DiD-labeled M@R and LysoTracker-stained lysosomes (Pearson's correlation coefficient: 0.5; Fig. 2f), indicating reduced lysosomal trafficking and potentially improved preservation of encapsulated R bioactivity. Together, these results support that macrophage membrane coating promotes the transcellular delivery of ranibizumab across ocular epithelial layers via intact vesicular transport.

To explore mechanisms underlying enhanced trans-barrier delivery, RNA sequencing was performed in HCE-T cells treated with M@R for 24 h. The distribution of differential-expression results is shown in Fig. 2g. Using |log_2_FC| ≥ 1 and adjusted *p* < 0.05 as thresholds, no differentially expressed genes were identified. In addition, immunofluorescence and Western blot analyses showed that neither R nor M@R noticeably altered expression of tight junction-related proteins in HCE-T cells (Fig. 2h and k). Pretreatment with endocytic inhibitors, including sucrose (energy-dependent uptake, 29.6% inhibition), chlorpromazine (clathrin-mediated, 51.2% inhibition), methyl-β-cyclodextrin (lipid raft–mediated, 45.2% inhibition), genistein (caveolae-mediated, 61.9% inhibition), and amiloride (macropinocytosis, 65.7% inhibition), significantly reduced cellular uptake of M@R (Fig. 2l and m) [34,35]. These findings suggest that M@R enters HCE-T cells via multiple energy-dependent pathways, including macropinocytosis and clathrin-/caveolae-mediated endocytosis, and traverses cellular barriers primarily through transcellular routes.

The RNA-seq results showed no differentially expressed genes after M@R treatment, implying that the enhanced transcellular transport does not depend on transcriptional remodeling of tight junctions or cytoskeletal components. This is consistent with the rapid onset of transcytosis, which is more likely governed by membrane protein-mediated endocytic pathways—as supported by our inhibitor experiments—rather than by slow gene expression changes. Thus, the enhanced uptake likely arises from the functional membrane proteins (e.g., adhesion molecules and receptors) on the macrophage membrane, which facilitate vesicular trafficking without altering the epithelial transcriptome.

### 2.3 *In vivo* and *ex vivo* analysis of M@R enhanced ranibizumab delivery across the corneal/scleral barrier to the fundus

**Fig. 3:**
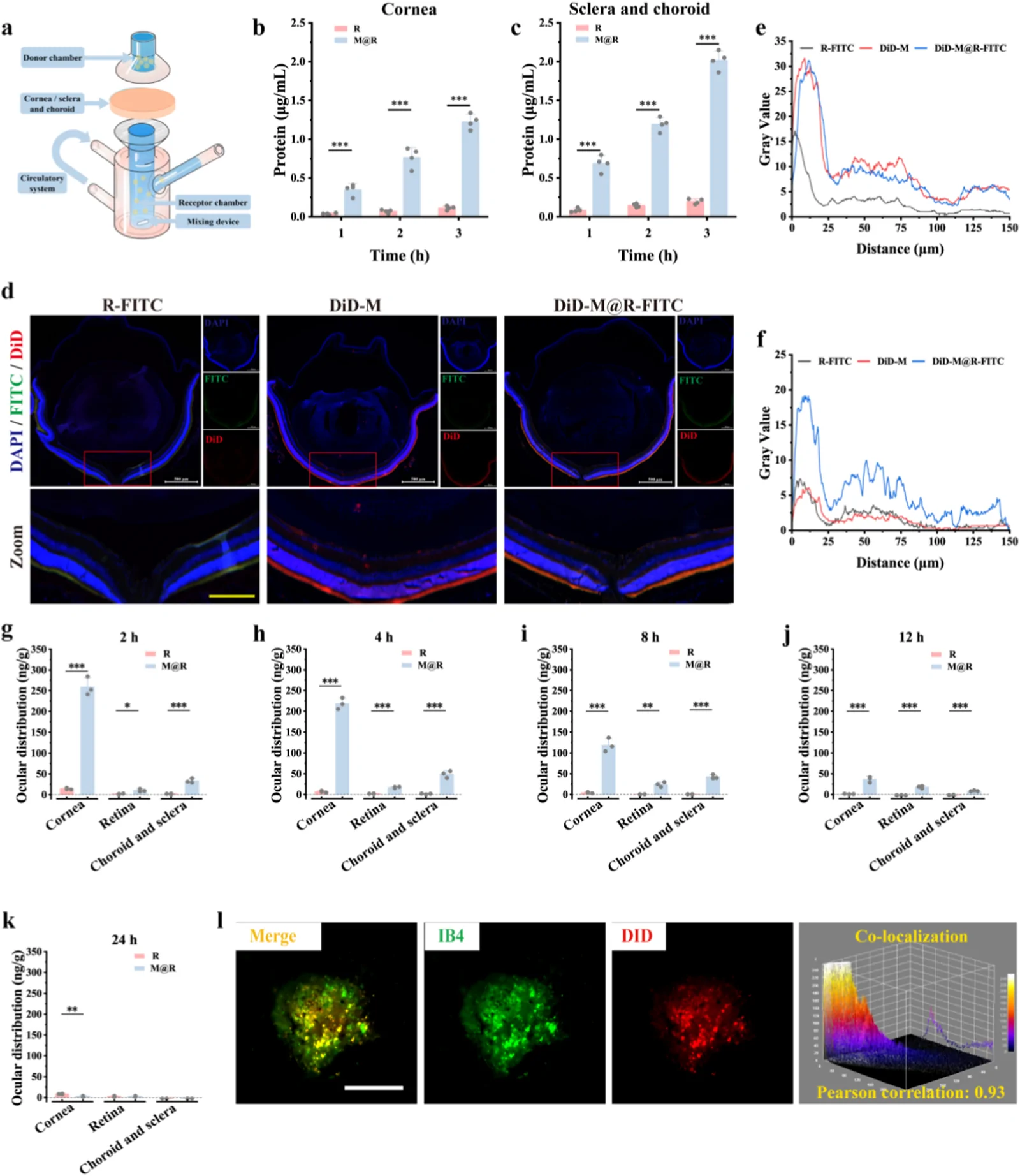
***Ex vivo* permeation and *in vivo* ocular distribution of M@R. (a)** Schematic representation of the Franz diffusion cell and assembly. Cumulative permeation of R and M@R across corneal **(b)** and sclera–choroid **(c)** tissues over time. **(d)** Representative fluorescence images showing distribution of R and M@R in retinal and choroidal tissues. Fluorescence intensity profiles of DiD **(e)** and FITC **(f)** from sclera to inner retina were quantified using ImageJ (scale bar, white: 500 μm; yellow: 200 μm). **(g-k)** ELISA quantification of ranibizumab concentrations in the cornea, retina, choroid and sclera at various time points after topical administration of R or M@R eye drops. **(l)** Representative images and colocalization analysis of DiD-M with CNV (scale bar, 100 μm). Data are presented as mean ± SD. Statistical analysis was performed using independent-samples T test or one-way ANOVA with multiple comparisons corrected by the Bonferroni test. *p < 0.05; **p < 0.01; ***p < 0.001.

To evaluate the enhanced tissue penetration of M@R compared to free R, an *ex vivo* Franz diffusion cell model was employed using isolated rabbit corneal and scleral-choroidal tissues [33]. Freshly excised tissues were mounted between the donor and receptor chambers (Fig. 3a). A 200 μL solution containing either free R or M@R was added to the donor chamber. Cumulative permeation of R through the tissues was quantified at various time points. M@R significantly enhanced penetration through both corneal and scleral-choroidal tissues compared with free R, whereas only minimal permeation was detected with free R (Fig. 3b and c). These data indicate a substantial improvement in delivery efficiency, supporting enhanced posterior delivery of R across corneal and scleral-choroidal barriers.

The ocular distribution of M@R after topical administration was assessed using fluorescent tracers in mice. R and M were labeled with FITC and DiD, respectively. At 6 h after topical administration, eyes were enucleated and cryosectioned for fluorescence microscopy. In the M and M@R groups, strong red fluorescence (DiD) was observed in the retina and choroid, with signal intensity decreasing from the sclera toward the retina (Fig. 3d and e). Compared with free R, the M@R group exhibited markedly stronger green fluorescence (FITC), showing a similar gradient (Fig. 3d and f).

Next, the ocular distribution of ranibizumab following topical administration of R and M@R was characterized using a ranibizumab ELISA kit. C57BL/6 mice received three consecutive topical applications of 5 μL R or M@R eye drops and were euthanized at predetermined time points (2, 4, 8, 12, and 24 h post-final dose). Corneal, retinal, and scleral-choroidal tissues were harvested and homogenized; ranibizumab concentrations in each tissue were quantified via ELISA (Fig. 3g–k). Following topical administration of free R eye drops, ranibizumab concentrations in all ocular tissues were either undetectable or extremely low at all time points examined. In striking contrast, after instillation of M@R eye drops, ranibizumab levels in the cornea peaked at 2 h post-dose (259.46 ± 22.53 ng/g) and subsequently declined gradually over time. Although initial drug concentrations in the posterior segment tissues were relatively low, they continued to rise progressively, reaching peak levels in the sclera-choroid at 4 h (48.98 ± 8.27 ng/g) and in the retina at 8 h (24.34 ± 5.70 ng/g) post-administration. Notably, the peak concentration in the sclera-choroid was substantially higher than that in the retina.

These findings indicate that M@R facilitates transocular delivery of ranibizumab to the posterior segment, primarily via the conjunctival-scleral-choroidal pathway, consistent with previously reported noninvasive strategies for macromolecular drug delivery to the fundus [[34], [35], [36]].

Chronic inflammation is a critical driver in the pathogenesis and progression of nAMD and DR [37,38]. To determine whether macrophage membrane vesicles retain inflammation targeting *in vivo*, we assessed inflammatory cytokines in a laser-induced CNV mouse model. Immunofluorescence revealed increased IL-1β and IL-6 signals at laser lesions in CNV mice compared with normal controls, indicating elevated local inflammation (Fig. S10). At 6 h after topical administration of DiD-M, pronounced red fluorescence accumulation was observed at the laser-induced lesions, with signal intensity significantly higher than that in the corresponding regions of control mice (60.91 ± 3.86 *vs.* 34.47 ± 0.90; *p* < 0.001), supporting preserved inflammation targeting of macrophage membranes *in vivo* (Fig. S11a and S11b). Furthermore, flat-mount immunofluorescence of the RPE-choroidal-scleral complex showed a high degree of colocalization between DiD-M (red) and IB4-labeled CNV (green) (Pearson's correlation coefficient: 0.93, Fig. 3l). Together, these results demonstrate that macrophage membrane vesicles can achieve targeted delivery to inflammatory CNV sites in the fundus.

### 2.4 Anti-angiogenic effects of M@R *in vitro*

The antiangiogenic potential of M@R was evaluated *in vitro* by assessing its effects on HUVEC proliferation, migration, and tube formation [[39], [40], [41]]. Untreated HUVECs served as the negative control, while VEGF was used to induce angiogenesis and simulate a pathological microenvironment. VEGF-stimulated HUVECs were then treated with R, M, or M@R.

**Fig. 4:**
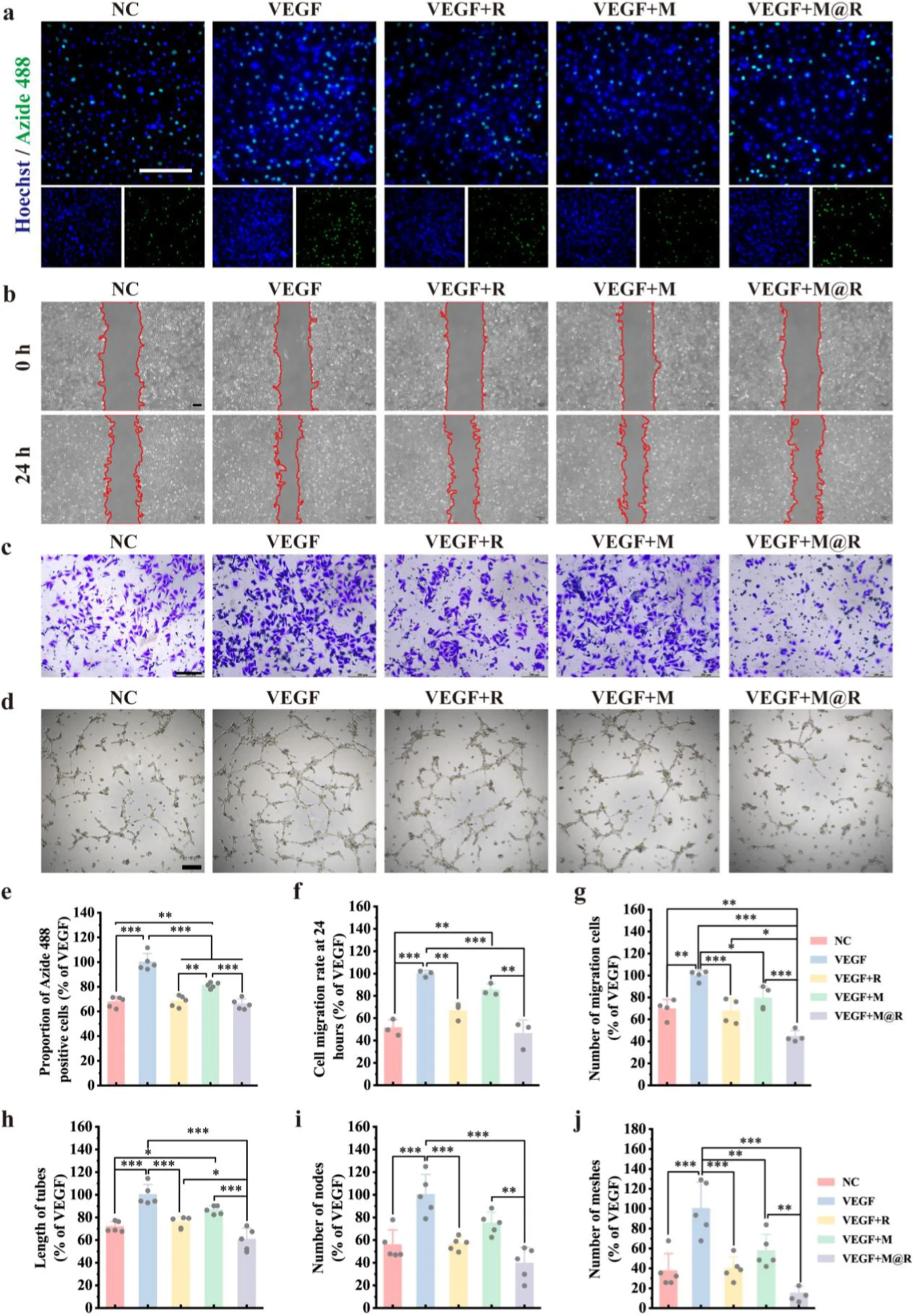
**Evaluation of the anti-angiogenic capacity of M@R *in vitro*. (a)** HUVEC proliferation measured by an EdU assay after 24 h of treatment with various formulations. The EdU-positive cell ratio was analyzed (green, EdU-positive cells; blue, nuclei). **(b)** Scratch assay of HUVECs after different treatments at 0 and 24 h. **(c)** Representative images of the HUVEC Transwell migration assay after 24 h of treatment with various formulations. **(d)** Tube formation assay of HUVECs after different treatments. **(e)** Quantitative analysis of the proliferating cell ratio. **(f)** Quantification of HUVEC migration after different treatments. **(g)** Quantitative analysis of migrated HUVECs in the Transwell assay. Quantitative analysis of total tube length **(h)**, node number **(i)**, and mesh number **(j)** in the HUVEC tube formation assay. Data are presented as mean ± SD. Statistical analysis was performed using one-way ANOVA with multiple comparisons corrected by the Bonferroni test. *p < 0.05; **p < 0.01; ***p < 0.001. Scale bar, 100 μm.

As shown in Fig. 4a and e, VEGF stimulation significantly enhanced HUVEC proliferation compared with the negative control, as indicated by an increased proportion of fluorescently stained cells. In contrast, treatment with R, M, or M@R significantly inhibited VEGF-induced proliferation, reducing the proportion of stained cells to approximately 68.25 ± 4.52%, 81.23 ± 2.40%, and 65.87 ± 4.28% of the VEGF group, respectively. These results indicate that M@R suppresses VEGF-driven HUVEC proliferation. HUVEC migration was assessed using scratch-wound and Transwell assays. VEGF markedly promoted migration, whereas both R and M@R attenuated this effect (Fig. 4b). Quantification showed migration rates of 66.38 ± 7.67% (R) and 46.01 ± 12.28% (M@R) relative to the VEGF group (Fig. 4f). Consistent results were obtained in the Transwell assay, in which M@R exhibited stronger inhibition than R alone (Fig. 4c and g). In Matrigel tube-formation assays, VEGF increased total tube length, node number, and mesh number, and these increases were suppressed by both R and M@R, with M@R showing greater inhibition across parameters (Fig. 4d, 4h–4j). Collectively, these findings indicate pronounced anti-angiogenic activity of M@R *in vitro*, which may be partly attributable to enhanced cellular uptake of R and stronger inhibition of VEGF signaling.

### 2.5 *In vivo* therapeutic efficacy analysis of M@R on CNV

**Fig. 5:**
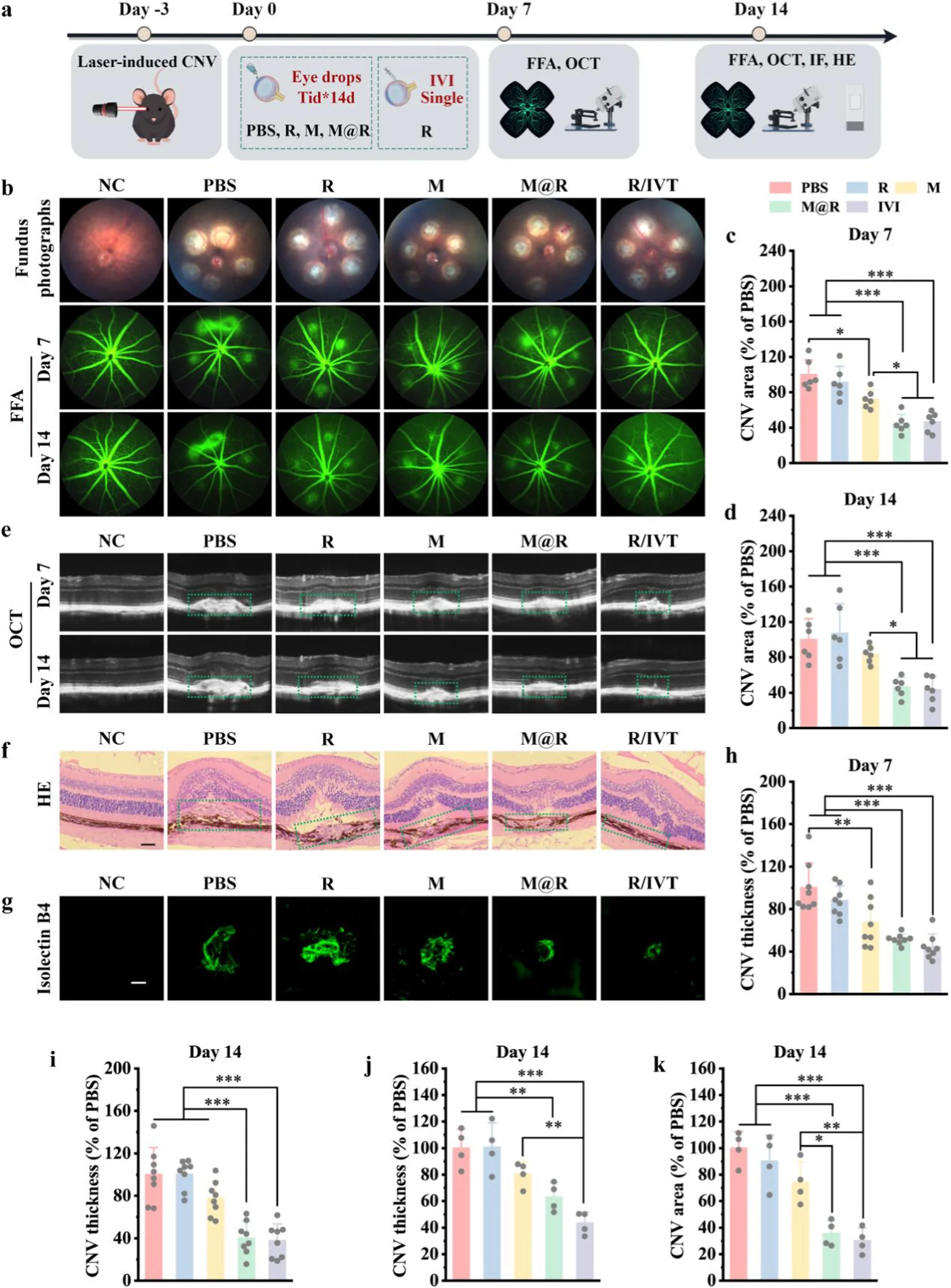
**M@R eye drops show therapeutic efficacy in a laser-induced CNV mouse model. (a)** Schematic illustration of laser-induced CNV model establishment and the *in vivo* experimental design. Experimental groups included normal controls and CNV mice treated with PBS, R, M, or M@R eye drops, or a single intravitreal injection of R. Topical formulations were standardized to R 1 mg mL^−1^, M 2.5 mg mL^−1^, and M@R 1 mg mL^−1^ (R-equivalent); intravitreal R was 10 mg mL^−1^. **(b)** Representative fundus photographs on day 0 and FFA images on days 7 and 14 after the indicated treatments. Quantitative analysis of relative fluorescence leakage area in CNV-bearing mice after 7 days **(c)** and 14 days **(d)** of treatment. **(e)** Representative OCT images on days 7 and 14 after the indicated treatments. **(f)** Representative H&E-stained retinal sections after 14 days of treatment. Scale bar, 100 μm. **(g)** Representative fluorescence images of RPE–choroid–sclera flat mounts stained with isolectin B4. Scale bar, 50 μm. Quantitative analysis of relative CNV thickness based on OCT images after 7 days **(h)** and 14 days **(i)** of treatment. **(j)** Quantitative analysis of relative CNV thickness based on H&E-stained sections after 14 days of treatment. **(k)** Quantitative analysis of relative isolectin B4-positive areas after 14 days of treatment. Data are presented as mean ± SD. Statistical analysis was performed using one-way ANOVA with multiple comparisons corrected by the Bonferroni test. *p < 0.05; **p < 0.01; ***p < 0.001.

Given the potent anti-angiogenic activity of M@R *in vitro*, we next evaluated its therapeutic effect on CNV via topical instillation in a laser-induced CNV mouse model, which has been widely used as a model of nAMD [31,34,40]. As outlined in Fig. 5a, after CNV induction, mice were randomly assigned to PBS eye drops, R eye drops, M eye drops, M@R eye drops (all administered three times daily for 14 days), or a single intravitreal injection of R (R/IVI).

Fluorescein fundus angiography (FFA) was performed on days 7 and 14 to assess vascular leakage, a marker of CNV activity (Fig. 5b). Quantitative analysis of leakage area revealed that, compared with PBS, neither R (91.17 ± 18.14% and 107.06 ± 33.32% of PBS on days 7 and 14, respectively) nor M (72.29 ± 10.41% and 83.36 ± 9.91% of PBS on days 7 and 14, respectively) eye drops significantly suppressed CNV. In contrast, both M@R eye drops and R/IVI demonstrated significant inhibitory effects, reducing the leakage area to approximately 44.32 ± 10.84% and 46.34 ± 11.16% of the PBS group value on day 7, and to 46.34 ± 10.97% and 43.40 ± 14.93% on day 14, respectively (Fig. 5c and d).

CNV thickness was evaluated by optical coherence tomography (OCT) on days 7 and 14 (Fig. 5e). Consistent with the FFA findings, R and M eye drops did not significantly reduce CNV thickness, except for a transient reduction observed with M on day 7 (67.50 ± 22.96% of PBS). Notably, M@R eye drops and R/IVI significantly decreased CNV thickness to 51.69 ± 4.95% and 44.47 ± 12.08% of PBS on day 7, and to 40.30 ± 15.62% and 37.65 ± 15.81% on day 14, respectively (Fig. 5h and i).

Histopathological examination was conducted at the end of treatment to corroborate therapeutic outcomes. H&E staining (Fig. 5f) showed that M@R eye drops and R/IVI significantly reduced CNV lesion size, reducing CNV thickness to 62.94 ± 10.72% and 43.30 ± 8.37% of PBS, respectively, whereas R or M eye drops showed no significant effect (100.58 ± 18.37% and 80.49 ± 9.28% of PBS, respectively; Fig. 5j). In addition, RPE-choroid-sclera flat mounts were stained with isolectin B4 to quantify CNV area (Fig. 5g). M@R eye drops and R/IVI reduced CNV area to 35.61 ± 9.71% and 30.21 ± 9.54% of PBS, respectively, whereas topical R or M showed minimal efficacy (90.22 ± 19.40% and 73.95 ± 15.95% of PBS, respectively; Fig. 5k).

**Fig. 6:**
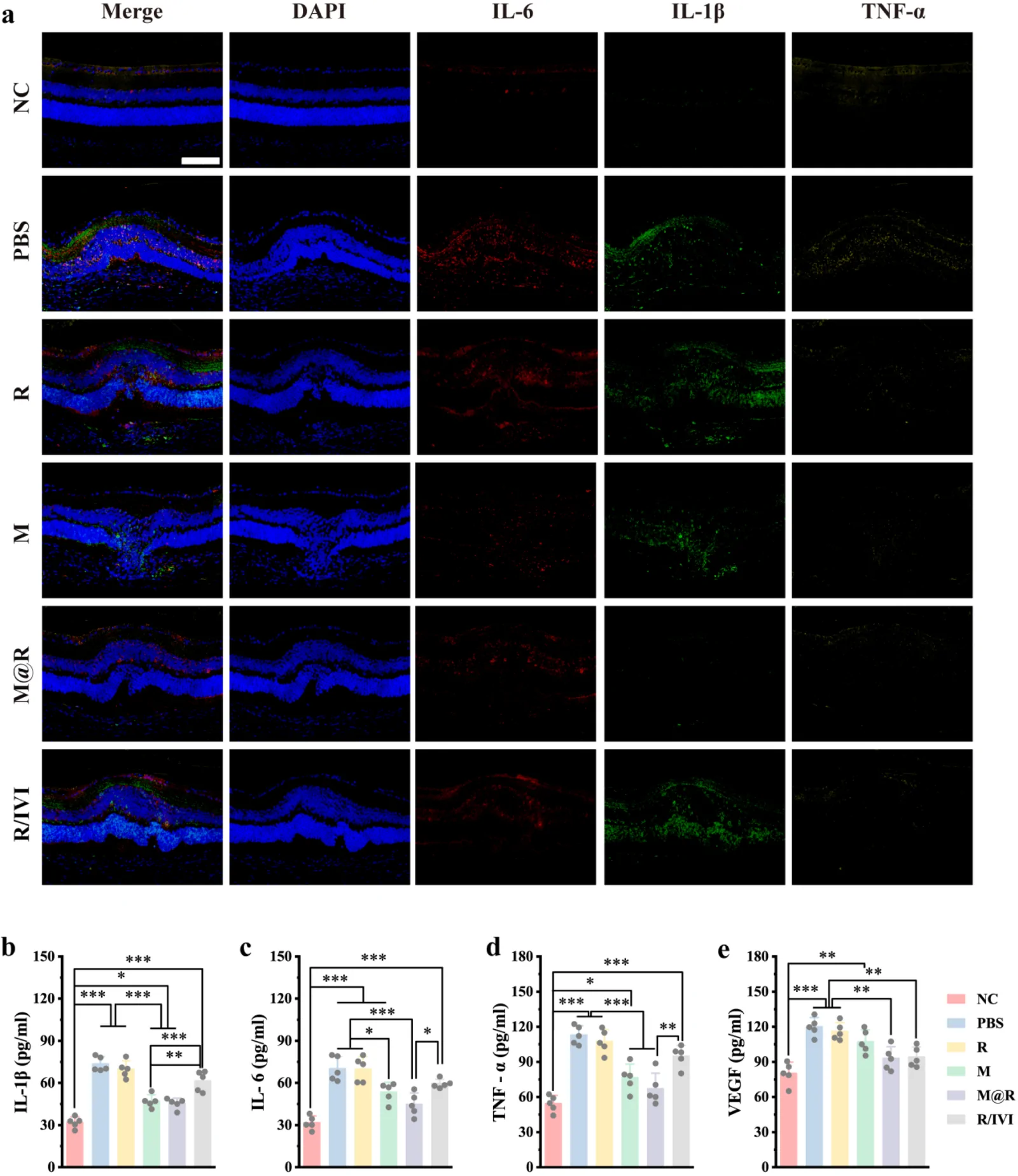
**M@R eye drops demonstrated anti-inflammatory and anti-VEGF effects in a laser-induced CNV mouse model. (a)** Representative immunofluorescence images of inflammatory factor levels in ocular sections after 14 days of various treatments. Quantitative analysis of IL-1β **(b)**, IL-6 **(c)**, TNF-α **(d)**, and VEGF **(e)** in retinal–choroidal tissues of mice with CNV, as determined by ELISA kits. Data are presented as mean ± SD. Statistical analysis was performed using one-way ANOVA with multiple comparisons corrected by the Bonferroni test. *p < 0.05; **p < 0.01; ***p < 0.001. Scale bar, 100 μm.

During the treatment period, the M eye drops group demonstrated a modest inhibitory effect on CNV, which may be partly attributable to anti-inflammatory properties of M via sequestration of inflammatory cytokines. After the treatment was completed, we measured the levels of cytokines in the eye cells of the mice. The results of immunofluorescence showed that the expression levels of inflammatory factors in the M and M@R eye drops groups were significantly reduced (Fig. 6a). The results of ELISA quantitative detection indicated that the expression levels of inflammatory factors and VEGF in the M@R group were both significantly decreased (Fig. 6b–e). Collectively, these findings indicate that despite the need for frequent instillation (three times daily for 14 days), M@R can inhibit the formation of CNV in laser-induced mice through inflammation-targeted accumulation, anti-inflammatory, and anti-VEGF synergistic effects, and its efficacy is comparable to that of intravitreal ranibizumab injection. However, it should be noted that although the murine CNV model is a well-validated preclinical surrogate for AMD in humans, direct translation of absolute efficacy metrics to the clinical setting warrants caution. The primary contribution of this study is the demonstration of the feasibility and therapeutic promise of a noninvasive, inflammation-targeted biomimetic ocular drug delivery strategy.

### 2.6 Biocompatibility analysis of M@R

To assess long-term biocompatibility of M@R, a 2-week safety study was conducted in mice. Healthy mice were treated topically with PBS, M, or M@R eye drops three times daily for 14 consecutive days. On day 7, fundus OCT was performed. On day 14, a comprehensive ocular examination was carried out, including slit-lamp biomicroscopy, corneal fluorescein staining, fundus photography, FFA, and OCT. After euthanasia, eyeballs and major systemic organs were harvested for histopathological evaluation via H&E staining to assess ocular and systemic biocompatibility.

**Fig. 7:**
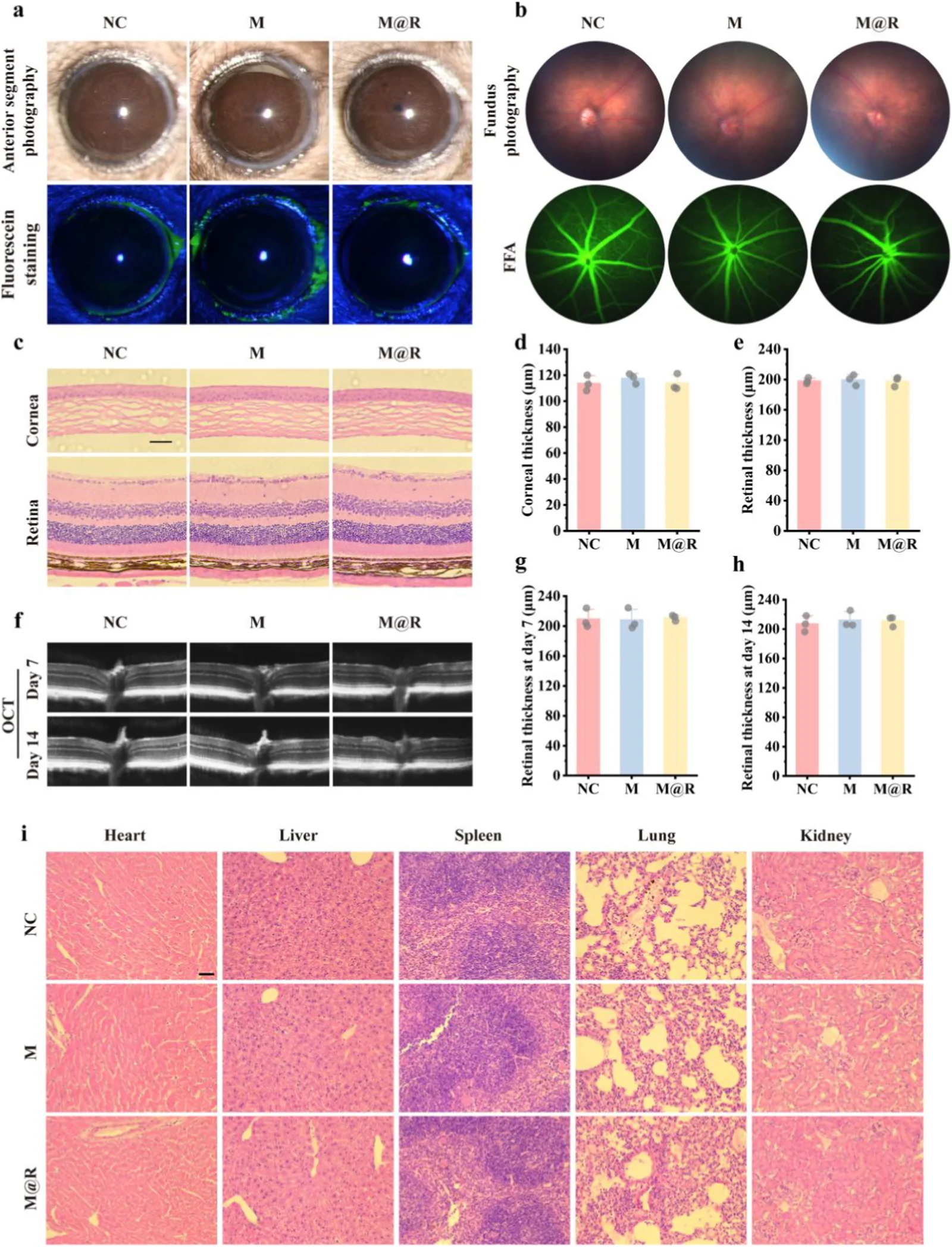
**Biocompatibility evaluation of M@R. (a)** Slit-lamp images of corneal fluorescein staining in mice treated with PBS, M, or M@R for 14 days. **(b)** Fundus photographs and FFA images in mice treated with PBS, M, or M@R for 14 days. **(c)** Representative H&E images of corneal and retinal tissues from mice treated with PBS, M, or M@R for 14 days. Quantitative analysis of corneal thickness **(d)** and retinal thickness **(e)** based on H&E-stained sections. **(f)** Representative OCT images of the retina after 7 and 14 days of treatment with PBS, M, or M@R. Quantitative analysis of retinal thickness at day 7 **(g)** and day 14 **(h)** based on OCT images. **(i)** Representative H&E images of major organs from mice treated with PBS, M, or M@R for 14 days. Data are presented as mean ± SD. Scale bar, 50 μm. Statistical analysis was performed using one-way ANOVA with multiple comparisons corrected by the Bonferroni test.

As shown in Fig. 7a, corneas in the M and M@R groups remained clear and transparent, comparable to controls, with no signs of ocular surface injury such as edema or epithelial defects. Fundus photography revealed no hemorrhages, edema, or retinal detachment in any group (Fig. 7b). FFA confirmed intact retinal vasculature without dye leakage (Fig. 7b). OCT imaging demonstrated preserved retinal layer architecture in all groups (Fig. 7f), with no significant differences in retinal thickness (Fig. 7g and h). H&E-stained sections revealed clear corneal and retinal structures without histopathological alterations such as edema or hemorrhage (Fig. 7c). ImageJ-based quantification of corneal and retinal thickness also showed no significant differences among groups (Fig. 7d and e). These results support favorable ocular biocompatibility of M@R.

Moreover, H&E staining of major systemic organs (heart, liver, spleen, lung, and kidney; Fig. 7i) showed no evident pathological changes, further supporting the long-term local ocular safety of M@R. In summary, the favorable biocompatibility profile and straightforward formulation support its translational potential.

## 3 Conclusions

In conclusion, we have developed a macrophage membrane–biomimetic nanovesicle encapsulating ranibizumab (designated M@R), which enables noninvasive, targeted delivery to inflamed tissues in the posterior segment of the eye. The biomimetic membrane coating facilitates transcytosis across ocular barriers and confers dual therapeutic functionality: selective homing to inflammatory foci and neutralization of pro-inflammatory cytokines. In a laser-induced CNV mouse model, topical administration of M@R achieved therapeutic efficacy comparable to that of intravitreal ranibizumab injection, attributable to synergistic anti-inflammatory and anti-VEGF effects. Furthermore, M@R exhibited favorable safety profiles both locally (ocular) and systemically. Collectively, this biomimetic nanoplatform constitutes a clinically translatable, noninvasive strategy for biologic therapy of posterior segment diseases, with the potential to reduce treatment burden and mitigate complications associated with repeated intravitreal injections.

## 4 Experimental section

### 4.1 Materials

Ethylisopropyl amiloride (EIPA, N132356), chlorpromazine hydrochloride (CPZ, C129569), methyl-β-cyclodextrin (MβCD, M102038), genistein (G106673), sucrose (S112234), fluorescein 5(6)-isothiocyanate (FITC, F106837), and recombinant mouse VEGF164 protein (rp154812) were purchased from Aladdin (USA). Ranibizumab was purchased from Novartis (USA). The following reagents were purchased from Beyotime (China): DiD (C1039), Membrane and Cytosol Protein Extraction Kit (P0033), Enhanced BCA Protein Assay Kit (P0009), LysoTracker Green (C1047S), EdU Cell Proliferation Kit with AF488 (C0071S), Immunostaining Fix Solution (P0098), Immunostaining Permeabilization Buffer with Saponin (P0095), Immunostaining Blocking Buffer (P0102), Crystal Violet Staining Solution (C0121), Coomassie Blue Super Fast Staining Solution (P0003FT), and Cell Counting Kit-8 (C0038). 4%–20% SDS-PAGE gels were purchased from ACE Biotechnology (China). ELISA kits for mouse IL-1β, IL-6, and TNF-α, and VEGF were purchased from Quanzhou Jiubang Biotechnology Co., Ltd. (China). ELISA kits for Ranibizumab were purchased from Jiangsu enzyme industry co., ltd., (China). Cytokines TNF-α (HY-P7090), IL-6 (HY-P7063A), and IL-1β (HY-P7073) were purchased from MedChemexpress (MCE, USA). Matrigel was purchased from Corning (USA). RIPA Buffer (R0010) and the Live/Dead Cell Double Stain Kit (CA1630) were purchased from Solarbio (China). IL-6R mouse mAb (222368), TNF Receptor 2 rabbit mAb (R30034), IL-1R2 rabbit pAb (680335), and Na^+^/K^+^ ATPase 1 rabbit mAb (R380790) were purchased from Chengdu Zhengneng Biotechnology Co., Ltd. (China). ZO-1 polyclonal antibody (21773-1-AP) and occludin polyclonal antibody (27260-1-AP) were purchased from Proteintech (China). Multiplex Immunofluorescence Staining Kit (G1236), Hoechst 33342 (G1127), DAPI (G1012), and FAS Eye Fixation Solution (G1109) were purchased from Servicebio (China).

### 4.2 Cell line and animals

Macrophages (RAW264.7), the human corneal epithelial cell line (HCE-T), retinal pigment epithelial cells (ARPE-19), and human umbilical vein endothelial cells (PUMC-HUVEC-T1) were obtained from Procell Life Science & Technology Co., Ltd. (Wuhan, China). RAW264.7 cells and HUVECs were cultured in RAW264.7 Cell Complete Medium and PUMC-HUVEC-T1 Cell Complete Medium (Procell Life Science & Technology Co., Ltd., Wuhan, China), respectively. HCE-T and ARPE-19 cells were cultured in DMEM/F-12 (Gibco) supplemented with 10% serum (Biochannel) and 1% penicillin–streptomycin (Gibco).

Male C57BL/6 mice (8–10 weeks old) were obtained from the Chongqing Medical University Laboratory Animal Center (Chongqing, China). All animal procedures were approved by the Institutional Animal Care and Use Committee (IACUC) of Chongqing Medical University (approval no. IACUC-CQMU-2025-12056). Animals were housed under standard conditions with a 12 h light/dark cycle and ad libitum access to food and water.

### 4.3 Synthesis and characterization of M@R nano-drops

*Synthesis of M@R nano drops:* Macrophage membranes were isolated from RAW264.7 cells using a Membrane and Cytosol Protein Extraction Kit (Beyotime, P0033) according to the manufacturer's instructions. Briefly, cells were washed with ice-cold PBS, resuspended in Reagent A, and incubated for 10 min. Two freeze–thaw cycles were then performed by alternating between liquid nitrogen and room temperature. The lysate was centrifuged at 700 × g for 10 min at 4 °C to remove nuclei and intact cells. The supernatant was collected and centrifuged at 14,000 × g for 30 min at 4 °C to pellet the membrane fraction. The supernatant was discarded, and the membrane pellet was resuspended in PBS and stored at 4 °C. For protein quantification, an aliquot of the membrane preparation was resuspended in Reagent B by vigorous vortexing for 5 s, followed by incubation on ice for 5 min; this process was repeated three times. After centrifugation at 14,000 × g for 5 min at 4 °C, the supernatant was collected, and the membrane protein concentration was determined using an Enhanced BCA Protein Assay Kit (Beyotime, P0009). The isolated macrophage membranes were mixed with ranibizumab (R) at a 1:1 (w/w) mass ratio (membrane: ranibizumab). Macrophage membrane–coated ranibizumab nano-drops (M@R) were prepared by sequential extrusion through 200- and 100-nm polycarbonate membranes using an extruder (Avanti, USA), followed by centrifugation at 100,000 × g for 30 min to remove unencapsulated ranibizumab. Membrane vesicles without ranibizumab (M) were prepared using the same procedure.

*Characterization of M@R:* The hydrodynamic diameter and zeta potential of M and M@R were measured by dynamic light scattering (DLS; Zetasizer). Particle morphology was examined by transmission electron microscopy (TEM; JEM-2100Plus, JEOL, Japan) after negative staining with uranyl acetate. UV–Vis spectra of M, R, and M@R were recorded from 200 to 350 nm using an ultraviolet–visible spectrophotometer. To assess stability, M@R was dispersed in PBS and stored at 4 °C, and particle size was monitored over time by DLS.

Protein profiles and retention of characteristic membrane proteins in M and M@R were evaluated by polyacrylamide gel electrophoresis. Total proteins were separated on 4%–20% SDS-PAGE gels (ACE Biotechnology) and visualized with Coomassie Blue Super Fast Staining Solution. For immunoblotting, proteins were transferred to PVDF membranes and then blocked with 5% skim milk. Membranes were incubated overnight at 4 °C with primary antibodies against TNF-αR2, IL-1βR, and IL-6R. After two washes with TBST, membranes were incubated with a horseradish peroxidase–conjugated secondary antibody. Signals were detected using a chemiluminescence system (Thermo Fisher). The cytokine-binding capacities of M and M@R were assessed using ELISA kits. Recombinant mouse IL-1β, TNF-α, and IL-6 were individually incubated with varying concentrations of M or M@R at 37 °C for 2 h. The mixtures were then centrifuged at 100,000 × g for 30 min, and the supernatants were collected. M@R was incubated with M1 macrophages for 24 h. Culture media were collected and centrifuged at 14,000 × g for 10 min. Concentrations of IL-1β, TNF-α, and IL-6 in the supernatants were quantified using the corresponding ELISA kits.

*Drug loading:* After extrusion, the resulting mixture was centrifuged at 100,000 × g for 30 min. The concentration of unencapsulated R in the supernatant was determined using an Enhanced BCA Protein Assay Kit (Beyotime, P0009). The amount of encapsulated R was calculated by subtracting the amount of R in the supernatant from the total amount initially added. Encapsulation efficiency (EE) and loading efficiency (LE) of M@R were calculated as follows:

EE = (the weight of R entrapped in M@R) / (total amount of R) × 100%.

LE = (the weight of R entrapped in M@R) / (the weight of M@R) × 100%.

### 4.4 M@R promotes transcellular delivery of ranibizumab *in vitro* and analysis of its mechanism

*In vitro cytotoxicity assessment:* HCE-T and ARPE-19 cells were seeded in 96-well plates (1 × 10^4^ cells per well) and incubated for 24 h to allow cell attachment. Cells were then treated with 100 μL of medium containing M or M@R at final concentrations of 0–40 μg mL^−1^. After 24 h, the medium was removed, and the cells were washed. Then, 100 μL of CCK-8 solution was added to each well. After incubation at 37 °C for 2 h, absorbance at 450 nm was measured using a microplate reader to assess cell viability. For live/dead staining, HCE-T cells were cultured in 24-well plates for 24 h and then incubated with M or M@R (1 mg mL^−1^) for an additional 24 h. Cells were then stained with calcein-AM and propidium iodide (PI) for 20 min and imaged using a fluorescence microscope (Leica, Germany). Live (green) and dead (red) cells were imaged using excitation at 488 and 561 nm, respectively.

*Cellular uptake:* Cellular uptake of M@R was assessed in HCE-T, ARPE-19, and HUVEC cells using an endocytosis assay. Cells were seeded in 24-well plates at 5 × 10^4^ cells per well and cultured for 24 h to allow attachment. Cells were then incubated with R-FITC or DiD-M@R-FITC at 10 μg mL^−1^ for the indicated time. Cellular internalization was visualized using a fluorescence microscope. In the acquired images, the FITC signal indicated the localization of R, the DiD signal represented the macrophage membrane, and Hoechst staining labeled cell nuclei.

*In vitro assessment of corneal epithelial penetration:* To evaluate whether M@R could penetrate the corneal epithelial barrier and deliver its payload to the basolateral compartment, an *in vitro* Transwell-based model was established. HCE-T cells were seeded onto Transwell inserts in 24-well plates and cultured for 7–10 days to form a confluent epithelial barrier. After the monolayer was established, 100 μL of R-FITC or M@R-FITC was added to the apical (upper) chamber. The system was then incubated at 37 °C for 12 h. Following incubation, absorbance of the medium in the basolateral (lower) chamber was measured at 494 nm using a microplate reader.

*Assessment of transcytosis:* Transcellular transport of M@R was evaluated in ARPE-19 and HCE-T cells using an endocytosis-based assay. Cells were seeded on glass coverslips in 24-well plates at 5 × 10^4^ cells per well and cultured for 24 h to allow attachment. Cells were then divided into two groups (Group 1 and Group 2). Group 1 was incubated with DiD-M@R-FITC (10 μg mL^−1^) for 12 h. After incubation, cells were washed and cultured for an additional 12 h in fresh complete medium without formulation. Cellular internalization and localization were then visualized by fluorescence microscopy. In the acquired images, the FITC signal indicated the distribution of R, the DiD signal represented the macrophage membrane, and Hoechst staining labeled cell nuclei.

*Lysosomal colocalization assay:* Cells were seeded in 24-well plates at 5 × 10^4^ cells per well and cultured for 24 h to allow attachment. Cells were then incubated with DiD-M@R (10 μg mL^−1^, R-equivalent) for 1 h to facilitate uptake. LysoTracker Green was then added at a final concentration of 50 nM and incubated for an additional 30 min to label lysosomes. Cells were subsequently imaged using a fluorescence microscope. Fluorescence signals from DiD (M@R) and LysoTracker Green (lysosomes) were acquired, and spatial overlap was analyzed. Colocalization between internalized M@R and lysosomal compartments was quantified using ImageJ.

*RNA Sequencing of HCECs:* HCE-T cells were seeded in T25 flasks and cultured to approximately 80% confluence. Cells were then incubated with M@R (10 μg mL^−1^) for 24 h; untreated cells served as the control. After treatment, cells were harvested, and total RNA was extracted for RNA sequencing.

*Immunofluorescence analysis of tight junction-related proteins:* HCE-T cells were seeded in 12-well plates at 2 × 10^5^ cells per well and cultured to form a confluent epithelial layer. Cells were then treated with R or M@R for 24 h. After treatment, cells were washed with PBS and fixed with Immunol Staining Fix Solution (Beyotime, P0098) for 15 min at room temperature. Following fixation, cells were permeabilized with Immunostaining Permeabilization Buffer with saponin (Beyotime, P0095) for 10 min and blocked with Immunol Staining Blocking Buffer (Beyotime, P0102) for 1 h at room temperature. Subsequently, cells were incubated overnight at 4 °C with primary antibodies against ZO-1 (1:200, Proteintech, 21773-1-AP) or occludin (1:200, Proteintech, 27260-1-AP). After washing three times with PBS, cells were incubated with Alexa Fluor 647–conjugated goat anti-rabbit IgG (H&L) secondary antibody (1:500, Beyotime, A0468) for 1 h at room temperature in the dark. Finally, cells were rinsed three times with PBST and counterstained with DAPI. Imaging was performed by fluorescence microscopy to visualize the localization of tight junction–related proteins.

*Western blotting analysis of tight junction-related proteins:* HCE-T cells were cultured to form a corneal epithelial layer and then treated with R or M@R for 24 h. Protein extracts were prepared using RIPA lysis buffer supplemented with 1× protease inhibitor cocktail (Beyotime). Protein concentrations were determined using a BCA Protein Assay Kit (Beyotime), and samples were diluted to equal concentrations using loading buffer (Beyotime). Next, proteins were separated on 4%–20% SDS-PAGE gels (ACE Biotechnology) and transferred to PVDF membranes (Merck Millipore). Membranes were blocked with 5% skim milk at room temperature for 1 h and incubated overnight at 4 °C with primary antibodies against ZO-1, occludin, and GAPDH (ZO-1 and occludin: 1:1000; GAPDH: 1:10,000; Proteintech). After washing with TBST, membranes were incubated with HRP-conjugated secondary antibodies at room temperature for 1 h. Signals were visualized using enhanced chemiluminescence (ECL) and imaged with a chemiluminescence system (Thermo Fisher). Band intensities were quantified using ImageJ and normalized to GAPDH.

*Flow cytometric analysis of endocytic pathways:* Flow cytometry was used to investigate endocytic mechanisms underlying M@R internalization in HCE-T cells. HCE-T cells were seeded in 12-well plates at 2 × 10^5^ cells per well and cultured for 24 h to allow attachment. To delineate uptake pathways, cells were preincubated for 1 h with pharmacological inhibitors before M@R treatment. Inhibitors were used at the following concentrations: 0.4 M sucrose, 10 μg mL^−1^ chlorpromazine, 2.5 mM methyl-β-cyclodextrin (MβCD), 0.2 mM genistein, and 0.5 mM amiloride. After inhibitor pretreatment, DiD-M@R was added at 10 μg mL^−1^ and incubated for 6 h. Cells were then detached using a trypsin-based dissociation solution, collected, and analyzed by flow cytometry. DiD fluorescence intensity was measured using a CytoFLEX flow cytometer and used as a readout of M@R internalization.

### 4.5 *In vivo* and *ex vivo* analysis of M@R enhanced ranibizumab delivery across the corneal/scleral barrier to the fundus

*Ex vivo corneal/scleral-choroidal permeability assay:* Fresh corneal or scleral–choroidal tissues were harvested from healthy adult New Zealand White rabbits after euthanasia by intravenous overdose of pentobarbital sodium. Corneal and scleral-choroidal tissues were mounted in separate Franz diffusion cells in independent experiments. Then, 0.2 mL of R or M@R (R final concentration: 1 mg mL^−1^) was added to the donor chamber, and the receptor chamber was filled with PBS. Aliquots (0.2 mL) were collected from the receptor chamber at 1, 2, and 3 h, and an equal volume of fresh PBS was immediately replenished. Ranibizumab (R) concentrations were quantified using an Enhanced BCA Protein Assay Kit.

*Ocular penetration and pharmacokinetics evaluation:* To evaluate ocular permeability, eye-drop formulations of R-FITC, DiD-M, and DiD-M@R-FITC were prepared. Each formulation was topically instilled into the conjunctival sac of mice (5 μL per eye, three consecutive instillations). At 6 h post-instillation, mice were euthanized; eyeballs were enucleated and fixed overnight in 4% paraformaldehyde. Eyes were cryoprotected in graded sucrose solutions until fully equilibrated, embedded in OCT compound, and snap-frozen in liquid nitrogen. Ocular cryosections (10 μm) were prepared, mounted with anti-fade medium containing DAPI, and imaged by CLSM to assess the ocular distribution of the fluorescent tracers. Similarly, R and M@R eye drops were administered topically to the mice (5 μL per eye, three consecutive instillations). Animals were euthanized at 2, 4, 8, 12, and 24 h post-final administration. Corneal, retinal, scleral and choroidal tissues were then harvested, and ranibizumab concentrations in each ocular tissue compartment were quantified using a commercially available ELISA kit, following the manufacturer's instructions.

### 4.6 Anti-angiogenic effects of M@R *in vitro*

*Cell proliferation assay:* HUVEC proliferation was assessed using an EdU Cell Proliferation Kit with Alexa Fluor 488. When HUVEC confluence reached ∼80%, cells were switched to FBS- and antibiotic-free medium. VEGF was applied to induce angiogenesis and simulate a pathological environment. Untreated HUVECs served as the normal control group. VEGF-stimulated HUVECs were then treated with PBS, free R, M, or M@R. The concentrations were standardized as follows: R at 10 μg mL^−1^, M at 25 μg mL^−1^, and M@R at 10 μg mL^−1^ (R-equivalent). After 24 h, cells were washed three times with PBS and incubated with EdU working solution. After 2 h, cells were fixed with 4% paraformaldehyde for 30 min and permeabilized with 0.3% Triton X-100 for 15 min. Cells were then blocked with 3% goat serum, followed by incubation with click-reaction buffer for 30 min at room temperature in the dark. Nuclei were counterstained with Hoechst 33342, and images were acquired by fluorescence microscopy.

*Wound healing assay:* HUVECs were seeded in 6-well plates at 4 × 10^5^ cells per well in 2 mL complete medium containing 10% FBS. After reaching >95% confluence, the monolayer was scratched using a sterile 200-μL pipette tip to create a wound across the center of the well. Cells were washed with PBS and then cultured with complete medium containing 2% FBS. VEGF was applied to induce angiogenesis and simulate a pathological environment. Untreated HUVECs served as the normal control group. VEGF-stimulated HUVECs were then treated with PBS, free R, M, or M@R. The concentrations were standardized as follows: R at 10 μg mL^−1^, M at 25 μg mL^−1^, and M@R at 10 μg mL^−1^ (R-equivalent). Wound closure was imaged at 0 and 24 h and quantified using ImageJ.

*Transwell assays for cell migration:* HUVEC migration was measured using Transwell chambers with an 8-μm pore size. Cells in FBS-free medium were seeded in the upper compartment at 1 × 10^4^ cells per well. The basolateral chambers were filled with 1 mL medium supplemented with 10% FBS to facilitate migration. VEGF-stimulated HUVECs were then treated with PBS, free R, M, or M@R. The concentrations were standardized as follows: R at 10 μg mL^−1^, M at 25 μg mL^−1^, and M@R at 10 μg mL^−1^ (R-equivalent). After 24 h, inserts were fixed with paraformaldehyde for 30 min and stained with crystal violet for 20 min. Non-migrated cells were removed from the upper surface using a cotton swab. Images were acquired by microscopy and quantified using ImageJ.

*Tube formation assay:* Tube formation assays were performed to investigate the effect of M@R on the tube-forming ability of HUVECs. When cell confluence reached approximately 80%, cells were transferred to 0.2% FBS medium for 1 d. Pre-chilled 96-well plates were coated with 50 μL Matrigel (8 mg mL^−1^), and HUVECs at a density of 4 × 10^4^ cells per well were seeded on the gel. VEGF was applied to induce angiogenesis and simulate a pathological environment. Untreated HUVECs served as the normal control group. VEGF-stimulated HUVECs were then treated with PBS, free R, M, or M@R. The concentrations were standardized as follows: R at 10 μg mL^−1^, M at 25 μg mL^−1^, and M@R at 10 μg mL^−1^ (R-equivalent). After 6 h, images were acquired by microscopy and analyzed using ImageJ.

### 4.7 *In vivo* therapeutic efficacy analysis of M@R on CNV

*Laser-induced CNV model:* Pupils were dilated using compound tropicamide eye drops (Shenyang Xingqi Pharmaceutical Co., Ltd., China), followed by intraperitoneal anesthesia with 1.25% tribromoethanol (0.2 mL/10 g, Nanjing Aibei Biotechnology Co., Ltd., China). A 532-nm laser (150 mW, 50 μm spot diameter, 100 ms) was used to induce photocoagulation at approximately 2 papillary diameters (PD) from the optic disc. Five laser burns were applied at equal distances around the optic nerve head in each eye. Bubble formation indicated rupture of Bruch's membrane. Three days post-induction, mice were randomized into five treatment groups: (1) PBS eye drops (5 μL per eye, three times daily for 14 days); (2) R eye drops (same dosing schedule); (3) M eye drops (same dosing schedule); (4) M@R eye drops (same dosing schedule); and (5) a single intravitreal injection of R (2 μL per eye, 10 mg mL^−1^). For topical dosing, formulations were standardized to contain R at 1 mg mL^−1^, M at 2.5 mg mL^−1^, and M@R at 1 mg mL^−1^ (R-equivalent).

*FFA and OCT:* FFA was conducted to evaluate retinal vascular permeability. Prior to imaging, pupils were dilated using compound tropicamide eye drops (Shenyang Xingqi Pharmaceutical Co., Ltd., China). Anesthesia was induced by intraperitoneal administration of 1.25% tribromoethanol (0.2 mL/10 g, Nanjing Aibei Biotechnology Co., Ltd., China). A 0.2 mL aliquot of 2% sodium fluorescein was injected intraperitoneally. Retinal images were captured using a Multi-Modal Ophthalmic Imaging System for Animals (Saris, Robotrak, China). Vascular permeability was analyzed based on the acquired images using ImageJ. OCT was subsequently performed for cross-sectional imaging of CNV lesions.

*Preparation and Analysis of RPE-Choroidal-Scleral Complex Flat Mounts:* Following FFA and OCT imaging, eyeballs were enucleated and fixed in eye fixation solution for 1 h. After washing with PBS, the RPE–choroidal–scleral complex was carefully dissected and blocked using the same buffer as described above for 2 h. Tissues were incubated overnight with FITC-conjugated isolectin B4. After thorough washing, samples were cut radially into petal-shaped segments and flat-mounted on glass slides. Images were acquired using a fluorescence microscope, and CNV lesions were quantified using ImageJ.

*Histological and Immunofluorescence Analysis:* Paraffin-embedded ocular sections were subjected to hematoxylin and eosin (H&E) staining. Briefly, enucleated eyes were fixed overnight in FAS fixative, embedded in paraffin, and sectioned at 5 μm. Sections were then stained with H&E, dehydrated through a graded ethanol series, cleared in xylene, and mounted with coverslips. Images were acquired using a Leica microscope (Leica Microsystems, Germany), and CNV thickness was quantified using ImageJ. For multiplex fluorescence detection, paraffin sections were processed using a commercial multiplex immunofluorescence staining kit according to the manufacturer's instructions. Sections were stained for the pro-inflammatory cytokines IL-1β, IL-6, and TNF-α. Imaging was performed using a confocal microscope to visualize and document colocalization of these markers.

*Cytokine quantification*: Following euthanasia, eyes were enucleated, and retinal–choroidal tissues were carefully dissected. Tissues were immediately rinsed with ice-cold PBS, blotted dry, weighed, and mechanically minced on ice. Subsequently, add PBS solution at a volume ninefold greater than the tissue weight and homogenize using a tissue homogenizer. The homogenate was centrifuged at 5000 × g for 10 min at 4 °C, and the clarified supernatant was collected for cytokine analysis. IL-1β, IL-6, TNF-α, and VEGF concentrations were determined using ELISA kits according to the manufacturer's instructions.

### 4.8 *In vivo* biocompatibility evaluation

To assess *in vivo* biocompatibility, mice received M or M@R via topical ocular instillation (three times daily for 14 consecutive days). On days 7 and 14, fundus OCT was performed. On day 14, additional examinations were conducted, including slit-lamp biomicroscopy for anterior segment imaging, fundus photography, and FFA. Following the final assessment, eyeballs and major systemic organs were harvested for histopathological sectioning and H&E staining.

### 4.9 Statistical analysis

All statistical analyses and graphical representations were performed using Origin 2024 software. Data are presented as mean ± SD. Comparisons between two groups were performed using an independent-samples *t*-test. Comparisons among multiple groups were analyzed by one-way ANOVA followed by a Bonferroni-corrected post hoc test. A two-sided *p* value < 0.05 was considered statistically significant, with *p < 0.05, **p < 0.01, and ***p < 0.001.

## CRediT authorship contribution statement

**Yongguo Xiang:** Data curation, Formal analysis, Investigation, Methodology, Visualization, Writing – original draft, Writing – review & editing. **Jiaojiao Kou:** Data curation, Methodology. **Huijie Cao:** Data curation, Validation. **Yuhui Zhang:** Methodology. **Hong Cheng:** Data curation. **Tu Wang:** Data curation. **Jialing Jiang:** Data curation. **Shuren Pan:** Data curation. **Juan Kang:** Data curation. **Yuanfu Ding:** Investigation. **Wenjuan Wan:** Supervision. **Shijie Zheng:** Supervision. **Man Li:** Funding acquisition, Project administration. **Xiaobei Huang:** Funding acquisition, Methodology, Supervision. **Ke Hu:** Funding acquisition, Project administration, Supervision.

## Ethics approval statement

All animal experimental protocols were approved by the Institutional Animal Care and Use Committee (IACUC) of Chongqing Medical University (approval no. IACUC-CQMU-2025-12056).

## Funding

This work was supported by the 10.13039/501100001809National Natural Science Foundation of China (Grant no. 82371098, 52403173), Genertec Medical Scientific Research Fund (Grant no. TYYLKYJJ-2025-029), 10.13039/501100005230Natural Science Foundation of Chongqing, China (CSTB2023NSCQ-MSX1077), and Chongqing Youth Innovation Talent Development Program (Grant no. CSTB2024NSCQ-QCXMX0094).

## Declaration of competing interests

The authors declare that they have no known competing financial interests or personal relationships that could have appeared to influence the work reported in this paper.

## Data Availability

Data will be made available on request.
