# Supplementary material for: Nano-drops of biomimetic macromolecular drugs based on macrophage membranes for targeted treatment of fundus diseases
Source: Mater Today Bio. 2026 Aug 19;40:103577. doi: 10.1016/j.mtbio.2026.103577 (PMC13528339; doi:10.1016/j.mtbio.2026.103577)
Supplement: Supplementary file 1 [file mmc1.docx]

**SUPPLEMENTARY MATERIALS**

**Nano-drops of Biomimetic Macromolecular Drugs Based on Macrophage Membranes for Targeted Treatment of Fundus Diseases**

Yongguo Xiang^1#^, Jiaojiao Kou^1,2#^, Huijie Cao^1#^, Yuhui Zhang^3#^, Hong Cheng^1^, Tu Wang^1^, Jialing Jiang^4^, Shuren Pan^5^, Juan Kang^5^, Yuanfu Ding^6^, Wenjuan Wan^1^, Shijie Zheng^1^, Man Li^2^*, Xiaobei Huang^3^*, Ke Hu^1,5^*

1. Department of Ophthalmology, The First Affiliated Hospital of Chongqing Medical University, Chongqing Key Laboratory for the Prevention and Treatment of Major Blinding Eye Diseases, Chongqing Eye Institute, Chongqing 400010, China.
2. Department of Ophthalmology, 363 Hospital, Chengdu 610041, China.
3. Chongqing Institute of Green and Intelligent Technology, Chinese Academy of Sciences, Chongqing 400714, China.
4. CQMU - University of Leicester Joint Institute, Chongqing Medical University, Chongqing 401331, China
5. Changdu People's Hospital of Xizang, Changdu 854085, China
6. School of Medical and Information Engineering, Scientific Research Center, Gannan Medical University, Ganzhou 341000, China

^#^ These authors contributed equally to this work.

* Corresponding author: Man Li, limxx@126.com; Xiaobei Huang, huangxiaobei@cigit.ac.cn and Ke Hu, cqhuke@hospital.cqmu.edu.cn.

**Figure S1.** Hydrodynamic particle size distribution and representative TEM images of M and M@R. Scale bar: 100 nm.

**Figure S2.** The hydrodynamic diameter distribution of M@R stored at 4℃ over time

**Figure S3.** The results of the enzyme-linked immunosorbent assay (ELISA) for the concentrations of IL-1β, IL-6 and TNF-α in the supernatant of the culture medium after co-incubation of M@R and M1 macrophages for 24 hours (n = 3). Data are presented as mean ± SD. Statistical analysis was performed using independent-samples T test. ****p* < 0.001.

**Figure S4.** The cytotoxicity of M and M@R to HCE-T and ARPE-19 cells at various concentrations. Cell viability of HCE-T after 24 h of incubation with M **(a)** and M@R **(b)**. **(c)** Representative images of live/dead cell staining after incubation of M and M@R at concentrations of 1mg/mL with HCE-T cells for 24 hours. Cell viability of ARPE-19 cells after 24 h of incubation with M **(d)** and M@R **(e)**. **(f)** Representative images of live/dead cell staining after incubation of M and M@R at concentrations of 1mg/mL with ARPE-19 cells for 24 hours. Scale bar: 200 μm.

**Figure S5.** Representative results of flow cytometry analysis at different time points after incubation of DiD-M@R-FITC with HCE-T cells.

**Figure S6.** Representative fluorescence images of R-FITC and DiD-M@R-FITC uptake by ARPE-19 cells at various time points. Scale bars: 200 μm.

**Figure S7.** Representative fluorescence images of R-FITC and DiD-M@R-FITC uptake by HUVECs at various time points. Scale bar: 200 μm.

**Figure S8.** Representative fluorescence co-localization images showing FITC (green channel) and DiD (red channel) signals, illustrating the time-dependent cellular uptake of DiD-M@R-FITC by HCECs, accompanied by quantitative Pearson correlation coefficient analysis. Scale bar: 50 μm.

**Figure S9.** Representative fluorescence co-localization images showing FITC (green channel) and DiD (red channel) signals, illustrating the intercellular transport of DiD-M@R-FITC between HCECs, accompanied by quantitative Pearson correlation coefficient analysis. Scale bar: 50 μm.

**Figure S10.** Representative images of immunofluorescence staining in ocular pathological sections from normal mice and mice with laser-induced CNV one week post-induction. Scale bar: 100 μm.

**Figure S11. (a)** Fluorescence representative images of ocular pathological sections of CNV mice and normal mice at 6 hours after local administration of DiD-M. **(b)** Quantitative measurements of mean fluorescence intensity in the choroid-retinal region of mice with CNV versus healthy control mice. Data are presented as mean ± SD. Statistical analysis was performed using independent-samples T test. ****p* < 0.001. Scale bar: 200 μm.
